# Susceptibility of HepG2 Cells to Silver Nanoparticles in Combination with other Metal/Metal Oxide Nanoparticles

**DOI:** 10.3390/ma13102221

**Published:** 2020-05-12

**Authors:** Sylwia Męczyńska-Wielgosz, Maria Wojewódzka, Magdalena Matysiak-Kucharek, Magdalena Czajka, Barbara Jodłowska-Jędrych, Marcin Kruszewski, Lucyna Kapka-Skrzypczak

**Affiliations:** 1 Centre for Radiobiology and Biological Dosimetry, Institute of Nuclear Chemistry and Technology, Dorodna 16, 03-195 Warsaw, Poland; m.wojewodzka@ichtj.waw.pl (M.W.); m.kruszewski@ichtj.waw.pl (M.K.); 2 Department of Molecular Biology and Translational Research, Institute of Rural Health, Jaczewskiego 2, 20-090 Lublin, Poland; magdalenamatysiak89@gmail.com (M.M.-K.); czajka.magdalenaa@gmail.com (M.C.); 3 Department of Histology and Embryology with Experimental Cytology Unit, Medical University of Lublin, Radziwiłowska 11, 20-080 Lublin, Poland; b.jedrych@gmail.com

**Keywords:** nanotoxicity, AgNP binary mixtures, neutral red assay, DNA damage, comet assay, reactive oxygen species, nanoparticle uptake

## Abstract

The fast-growing use of nanomaterials in everyday life raises the question about the safety of their use. Unfortunately, the risks associated with the use of nanoparticles (NPs) have not yet been fully assessed. The majority of studies conducted so far at the molecular and cellular level have focused on a single-type exposure, assuming that NPs act as the only factor. In the natural environment, however, we are likely exposed to a mixture of nanoparticles, whose interactions may modulate their impact on living organisms. This study aimed to evaluate the toxicological effects caused by in vitro exposure of HepG2 cells to AgNPs in combination with AuNPs, CdTe quantum dot (QD) NPs, TiO_2_NPs, or SiO_2_NPs. The results showed that the toxicity of nanoparticle binary mixtures depended on the type and ratio of NPs used. In general, the toxicity of binary mixtures of NPs was lower than the sum of toxicities of NPs alone (protective effect).

## 1. Introduction

Nanoparticles (NPs) are defined as a material that has all dimensions less than 100 nm. Chemical, physical, and biological properties of NPs are determined by their size, shape, structure, composition, and surface coating and usually differ from those of the bulk material of the same form [1]. Unique physicochemical properties make NPs a superior material, indispensable in numerous commercial and medical applications. In addition, NPs easily penetrate the human body and cross all-natural barriers [2]. However, the same properties that make NPs beneficial for society raise concerns about their potential toxicity to cells, organisms, and the environment.

In recent years, numerous scientific studies have been focused on the identification of expressed toxicity of different NPs. Many studies have shown detrimental effects that NPs exert on cells, tissues, and whole organisms. The toxicity of silver NPs (AgNPs), cadmium–tellurium quantum dots (CdTeQDs), and silica NPs (SiO_2_NPs) has been described by us and others several times. AgNPs decreased the viability and proliferative potential of many cell types in culture, including human liver carcinoma HepG2 cells [3,4,5,6]. CdTeQDs also express significant toxicity, attributed mostly to their heavy metal components [7]. However, some data also indicate that there is no visible evidence of cell wall integrity interruption or changes in cell numbers after treatment of different QDs [8]. To evaluate the toxicity of SiO_2_NPs to the HepG2 cell line, NPs of different sizes have been used at a range of concentrations and treatment times. The results indicated a decrease in cell viability in a time- and dose-dependent manner. Besides, the cytotoxicity of SiO_2_NPs exhibited an apparent NP size dependence [9,10]. On the other hand, some results showed negligible cytotoxicity of SiO_2_NPs [11,12]. Studies on the toxicity of titanium oxide NPs (TiO_2_NPs) also gave inconsistent results, likely reflecting the complexity of the problem. TiO_2_NPs have two crystalline forms, anatase and rutile, of which the anatase form is more biologically active [3]. TiO_2_NPs decreased the viability and mitochondrial succinate dehydrogenase activity of HepG2 cells in a concentration and time-dependent manner [13,14]. On the contrary, gold NPs (AuNPs) have been considered as inert and biocompatible, having negligible toxicity. However, intense studies on AuNPs resulted in an increasing number of reports on their potential toxicity on HepG2 cells [15,16].

The vast majority of these studies, however, have focused on the individual type of NPs. By contrast, the rapid development of nanotechnology increases the possibility that different types of nanomaterials could be found in the same medium (water, air, ground, food) and affect living organisms simultaneously. As there is a lack of systematic studies on this topic, in this study, we aimed to investigate the toxicity of AgNPs, renowned for their toxicity, in combination with AuNPs, CdTeQDs, SiO_2_NPs, and TiO_2_NPs. Different endpoints were used, such as binding/uptake, viability and metabolic activity, generation of reactive oxygen species (ROS), and genotoxicity.

## 2. Materials and Methods 

### 2.1. Nanoparticle Preparation and Characterization

Spherical AgNPs of nominal size 20 ± 5 nm and CdTeQDs of nominal size 3.8 nm (emission 650 nm ± 5 nm) were purchased from PlasmaChem (Berlin, Germany). The anatase/rutile TiO_2_NPs of nominal size 21 nm were purchased from Degussa-Evonik (Essen, Germany). SiO_2_NPs of nominal size 25 nm were purchased from Sigma Aldrich. AuNPs of nominal size 20 nm were obtained from NanoComposix (Praha, Czech Republic).

Stock solutions of AgNPs, TiO_2_NPs, SiO_2_NPs, and CdTeQDs were prepared as previously described [17]. In brief, nanoparticles (2 mg) were suspended in 800 μL of distilled water and sonicated on ice with a dose of 4.2 kJ/cm^3^. One hundred microliters of 15% bovine serum albumin and 100 μL of 10x concentrated phosphate-buffered saline (PBS) were added immediately after sonication. Stock solutions of all tested NPs were prepared and sonicated before each experiment.

The hydrodynamic diameter and zeta potential (ζ) were measured by dynamic light scattering (DLS) using a Zestasizer Nano ZS system (Malvern, UK) at 25 °C with a scattering angle of 173^°^. Stock solutions were diluted 1:8 in a full culture medium and measured in triplicate with 14 sub runs. The suspensions had pH = 7.4. Zeta potentials were calculated using the Smoluchowski limit for the Henry equation with a setting calculated for practical use (f(ka) = 1.5).

### 2.2. Cell Culture

Human hepatic cell line HepG2 was purchased from the American Type Culture Collection (ATCC, Rockville, MD, USA) and maintained according to the ATCC protocol. Briefly, cells were cultured in EMEM medium supplemented with 10% fetal calf serum (Gibco). The cells were incubated in a 5% CO_2_ atmosphere at 37 °C.

The HepG2 cell line is the most frequently used hepatoma cell line for studying hepatoxicity. The cells are used in the initial screening of toxicity in drug testing, and recently especially in nanotoxicology [18,19].

### 2.3. Flow Cytometry Evaluation of Cellular Binding/Uptake of Nanoparticles by Cells

The binding/uptake of NPs by HepG2 cells was examined by flow cytometry. The approach is based on the measurement of side scatter (SSC) values of the cell population [20]. In brief, twenty-four hours after the cells were seeded onto 6-well plates, nanoparticles were added for the next four hours of incubation. After the treatment, cells were washed two times with PBS to remove loosely bound particles, detached with the use of trypsin-EDTA, and spun down. The cell pellet was resuspended in 1 ml PBS for flow cytometry. The analysis was performed on a BD LSR Fortessa cytometer (BD Biosciences) equipped with a 488 nm laser, FSC diode detector, and photomultiplier tube SSC detector. Following the gating on the FSC vs. SSC chart, control and particle-exposed cells were run at low flow rates. Data from 20,000 events per sample were stored. The increase in SSC was calculated by dividing the mean SSC value of the particle-treated cells by the mean SSC value of control cells.

### 2.4. Cytotoxicity Evaluation

The impact of NPs alone or their mixtures on the proliferation of HepG2 cells was measured using a Neutral Red (NR) assay. The test was performed as described in Lankoff et al. [17]. In brief, HepG2 cells were seeded in 96-well microplates (TPP, Switzerland) at a density of 1x10^4^ cells/well in 100 μL of culture medium. Twenty-four hours after seeding, cells were treated with AgNPs, CdTeQDs, AuNPs, TiO_2_NPs, and SiO_2_NPs alone or mixtures of AgNPs and CdTeQDs, AuNPs, TiO_2_NPs, or SiO_2_NPs at concentrations of 10 or 50 µg/mL for 48 h. After treatment, cells were incubated for three hours at 37 °C with 100 µL of NR solution (5 mg/mL of NR in PBS was diluted 1:100 in the cell culture medium, incubated for 12 h at 37 °C, and centrifuged to remove any undissolved NR powder). Next, NR solution was aspirated, cells were washed with 150 µL of PBS, and 150 µL of an acid-ethanol solution (49% water, 50% ethanol, and 1% acetic acid) was added to each well. After 15 min of gentle shaking, the optical density was read at 540 nm in the plate reader spectrophotometer Infinite M200 (Tecan, Austria). At least three independent experiments in six replicate wells were conducted per concentration.

### 2.5. Measurements of Reactive Oxygen Species Formation

Intracellular ROS were measured based on the intracellular peroxide-dependent oxidation of 2′,7′-dichlorodihydrofluorescein diacetate (DCFH-DA; Sigma Aldrich) to form the fluorescent compound 2′,7′-dichlorofluorescein (DCF). Cells were seeded onto 6-well plates at a density of 2.5 × 10^5^ cells per well and cultured for 24 h. After washing with PBS, fresh medium containing AgNPs, CdTeQDs, AuNPs, TiO_2_NPs, and SiO_2_NPs alone or the mixtures of CdTeQDs, AuNPs, TiO_2_NPs, or SiO_2_NPs with AgNPs were added, and the cells were incubated for 2 h. After incubation, the treated cells were trypsinized, resuspended in the fresh medium, and the DCFH-DA solution was added to the final concentration of 5 µM. After 30 min incubation with the dye, forward scatter (FCS), side scatters (SSC) parameters, and the fluorescence of cells were measured in a BD LSR Fortessa cytometer (BD Biosciences).

### 2.6. Alkaline Comet Assay

The comet assay (single-cell gel electrophoresis) was performed as described in [21]. Briefly, an aliquot of cell suspension was mixed with an equal volume of 2% low-melting-point agarose (Type VII, Sigma), put on a microscope slide pre-coated with 0.5% normal agarose (Type IA, Sigma) and left on the ice. After agarose solidification, the slides were immersed in ice-cold-lysis solution (2.5 M NaCl, 100 mM Na2EDTA, 10 mM Tris, and 1% Triton X-100, pH 10). After 1 h lysis, the slides were placed on a horizontal gel electrophoresis unit filled with a fresh electrophoretic buffer (1 mM Na_2_EDTA (sodium ethylenediamine tetraacetate) and 300 mM NaOH) and allowed to stay in the buffer for 40 min for DNA unwinding. Next, electrophoresis was a performer (1.2 V/cm, 30 min, 10 °C). After electrophoresis, the slides were washed with 0.4 M Tris, pH 7.5 (3 × 5 min) and stained with 4,6-diamidino-2-phenylindole (DAPI), 50 μL per slide (1 µg/mL). The same procedure was applied for the measurement of DNA base damage. The treated cells were incubated on slides with the formamido-pyrimidine DNA glycosylase (FPG, New England BioLabs, UK), as described in [22]. Briefly, after lysis, the slides were washed 3× at 5 min each with the FPG buffer (40 mM Hepes (4-(2-hydroxyethyl)-1-piperazine ethanesulfonic acid), 0.1 M KCl, 0.5 mM EDTA, 0.2 mg/mL bovine serum albumin, pH 8) at 4 °C. Further, 50 μL of FPG solution (4.8 × 10^–2^ U) in the buffer was placed on each slide, covered with cover glass and incubated for 30 min in a light-protected box at 37 °C. The slides were stained with DAPI (1 µg/mL) and analyzed as described above. Image analysis of the data was performed with the Comet Assay IV Image Analysis System (Perceptive Instruments, UK). Fifty randomly selected comets per slide were analyzed, two slides per experimental point. The percentage of DNA in the comet tail was used in this study as a measure of DNA damage. 

### 2.7. The Concept of “Expected Value”

The measured toxicity, induction of DNA damage, or uptake/binding of NP mixtures was compared to the “expected value.” The “expected value” concept is based on the assumption that the action of both factors (two types of NPs) is independent, and their combined toxicity is a sum of toxicities of each factor alone (neutral or additive effect). If the combined toxicity is lower than the ”expected value”, the sparring (antagonistic) effect is observed. If the combined toxicity is higher than the ”expected value,” the synergistic (potentiating) effect is observed.

### 2.8. Statistical Analysis

At least three independent experiments were conducted for each experimental point. If results are expressed as the percentage of the untreated control, statistical evaluation of their significance was done on raw data. The difference between samples and the control was evaluated using GraphPad Prism 5.0 software (USA). Data were assessed by Kruskal–Wallis One Way Analysis of Variance on Ranks (ANOVA) followed by the post-hoc Dunnet’s method. Differences were considered statistically significant when the *P*-value was <0.05.

## 3. Results

### 3.1. Nanoparticles Characterization

Characterization of physical properties revealed that the hydrodynamic diameter of all NPs was significantly larger than their nominal size declared by the manufacturer. It is usually an observed phenomenon probably associated with the formation of protein corona on the surface of NPs, resulting in the apparent increase in size. The hydrodynamic diameter of binary mixtures of NPs as compared to single ones showed an increase in hydrodynamic diameter and zeta potential, as well as the polydispersity index (PDI) (Table 1). To further characterize NPs alone or in the mixture, their agglomeration in culture media was studied over 120 min. Among the studied NPs, only AgNPs and CdTeQDs showed marked agglomeration. AgNPs doubled their diameter over 30 min, whereas CdTeQDs agglomerated even faster and doubled their diameter over 15 min (Table 2). The initial hydrodynamic diameter of the NPs in mixture roughly corresponded to the sum of single NPs. Interestingly, the agglomeration of AgNPs in the mixture with other NPs is much slower than AgNPs alone. None of the AgNP mixtures doubled their initial diameter over 120 min; however, the PDI of mixtures indicated a high diversity of NP sizes (Table 3).

### 3.2. Flow Cytometry Evaluation of Cellular Binding/Uptake of NPs

**Figure 1:**
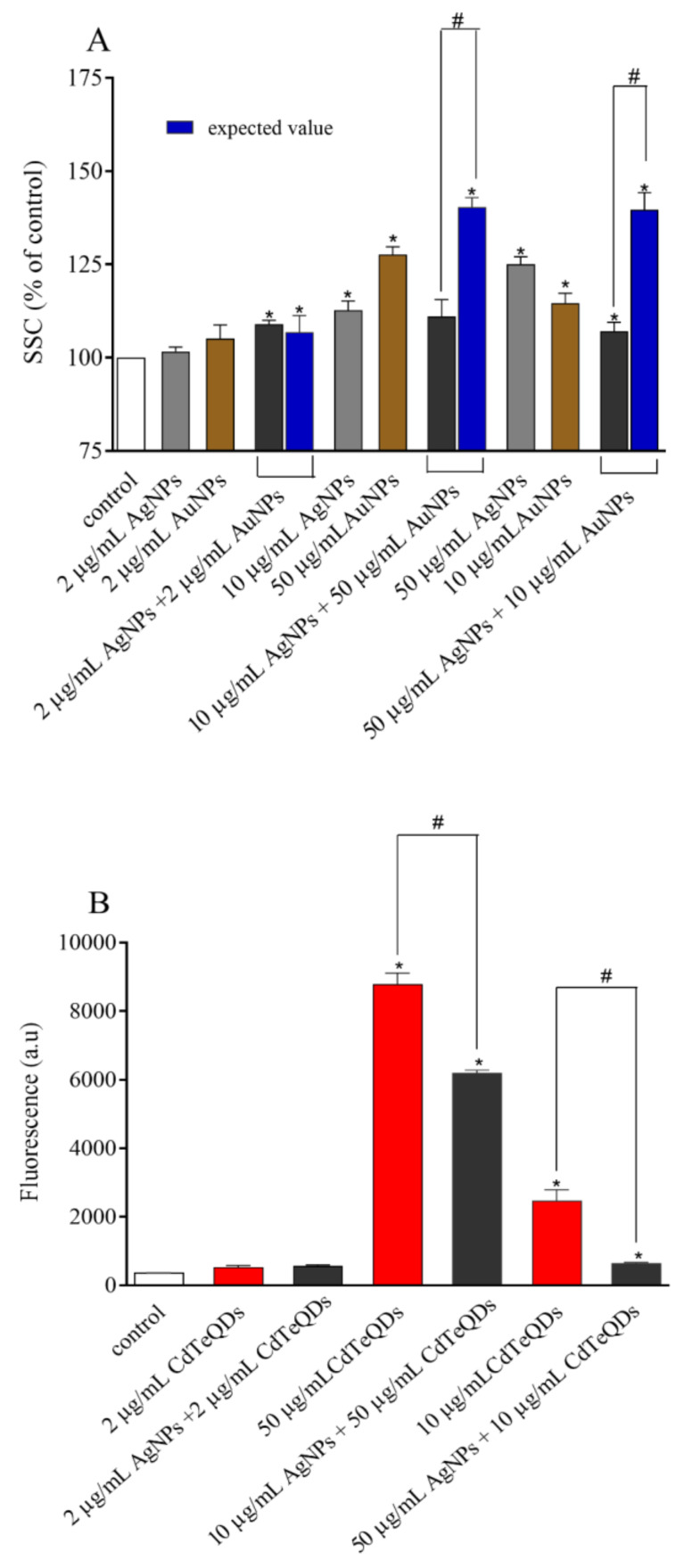

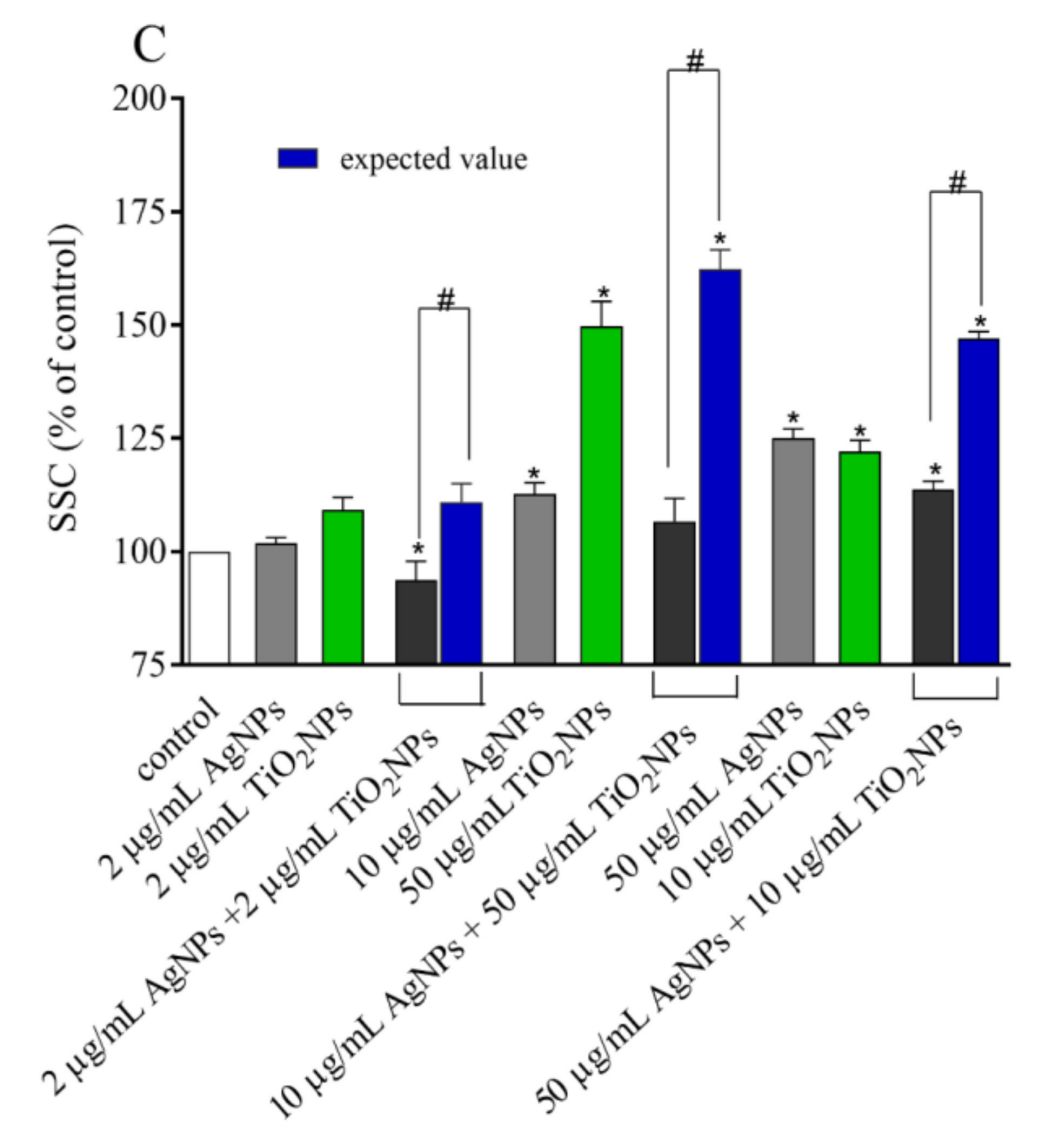

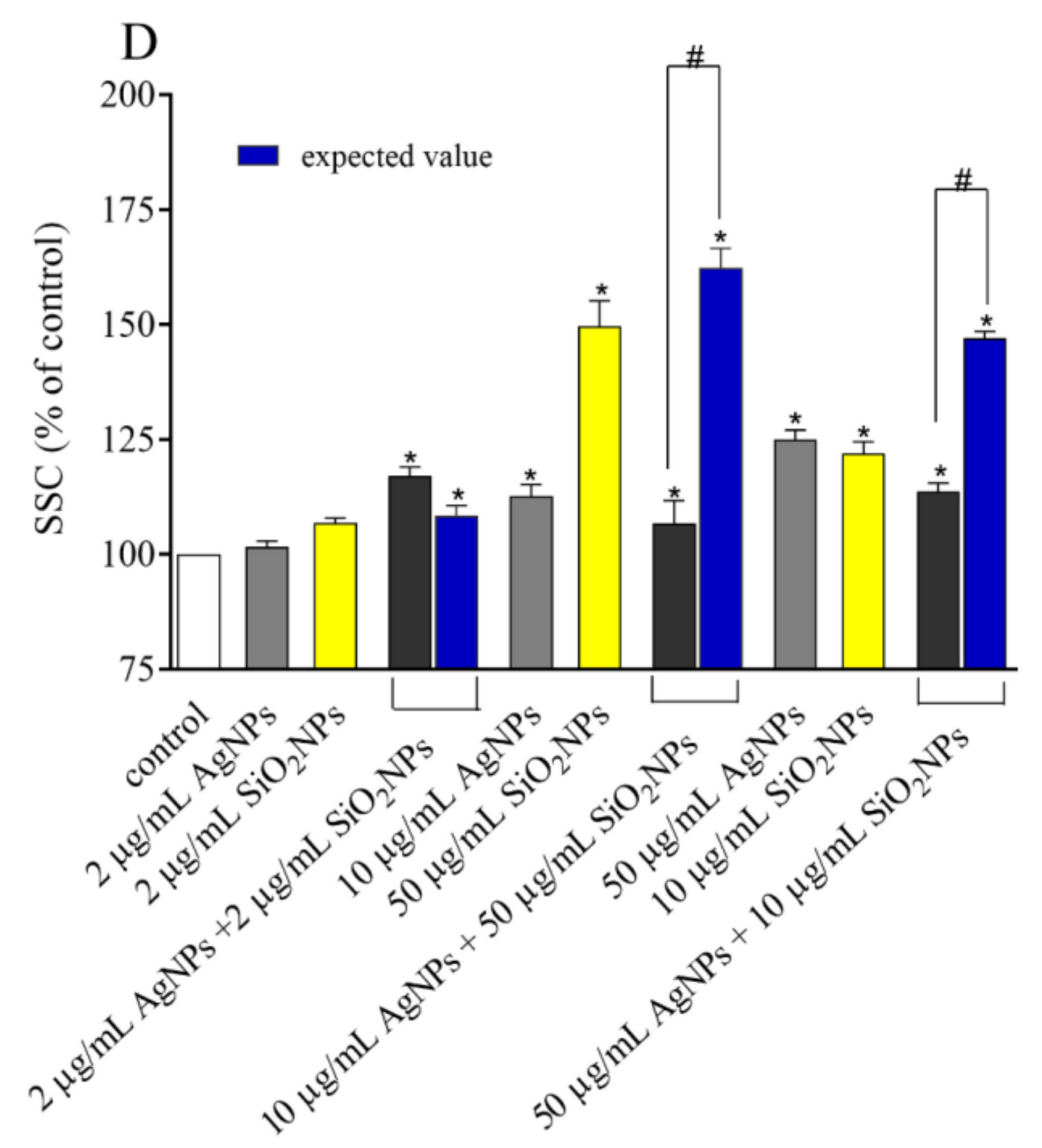
Uptake/binding of the single NPs or combination of AgNPs with (**A**) AuNPs, (**B**) CdTe quantum dots (QDs), (**C**) TiO_2_NPs, and (**D**) SiO_2_NPs. Means ± SD, n = 3. * denotes a statistically significant difference from the unexposed control, Student’s t-test applied to raw data, *P* < 0.05. # denotes a statistically significant difference between the observed and expected value, Student’s t-test applied to raw data, *P* < 0.05.

Uptake and/or binding of NPs to HepG2 cells was estimated from SSC values (for metallic NPs) or fluorescence (for CdTeQDs) measured by flow cytometry. After 4 h of incubation, all studied NPs showed a dose-dependent increase in uptake/binding. However, when observed values for NP mixtures were compared to the expected ones (a sum of uptake of single NPs), the NP uptake was significantly lower in all cases, except for treatment with AuNPs + AgNPs, CdTeQDs + AgNPs, and SiO_2_NPs + AgNPs all in concentrations of 2 μg/mL (Figure 1).

### 3.3. Cytotoxicity

The cytotoxicity of AgNPs, AuNPs, and CdTeQDs in the HepG2 cellular model has been described by us [23]. To keep the paper consistent, we recall these data in the Supplementary Figure S1, together with new data on the toxicity of TiO_2_NPs and SiO_2_NPs. As individuals, AgNPs and CdTeQDs have proven to be the most cytotoxic NPs. After treatment of the cells with AgNPs, a significant decrease in survival after 48 h of incubation was observed. IC_50_ for AgNPs after 48 h of incubation was approx. 45 μg/mL. CdTeQDs have proven to be even more toxic than AgNPs. The treatment with these NPs in the concentration range of 5–50 µg/mL also resulted in a significant decrease in survival. IC_50_ for CdTeQDs after 48 h of treatment was approx. 3 μg/mL. At the highest concentration tested, only 2% of cells survived after 48 h incubation as compared to the control. Other tested nanoparticles (TiO_2_NPs, SiO_2_NPs, and AuNPs) were less or not toxic for HepG2 cells.

**Figure 2:**
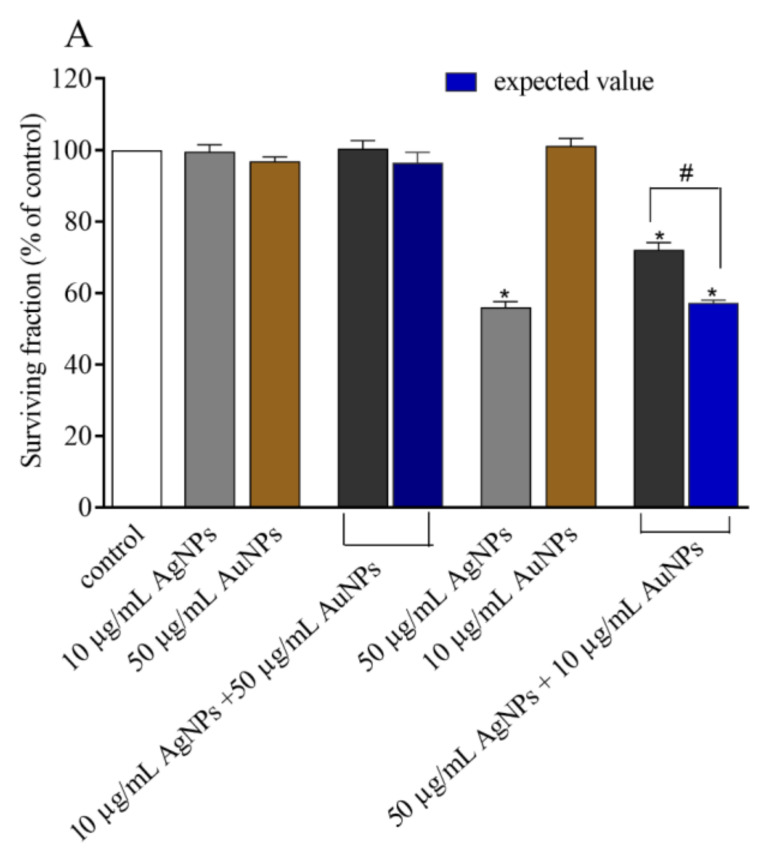

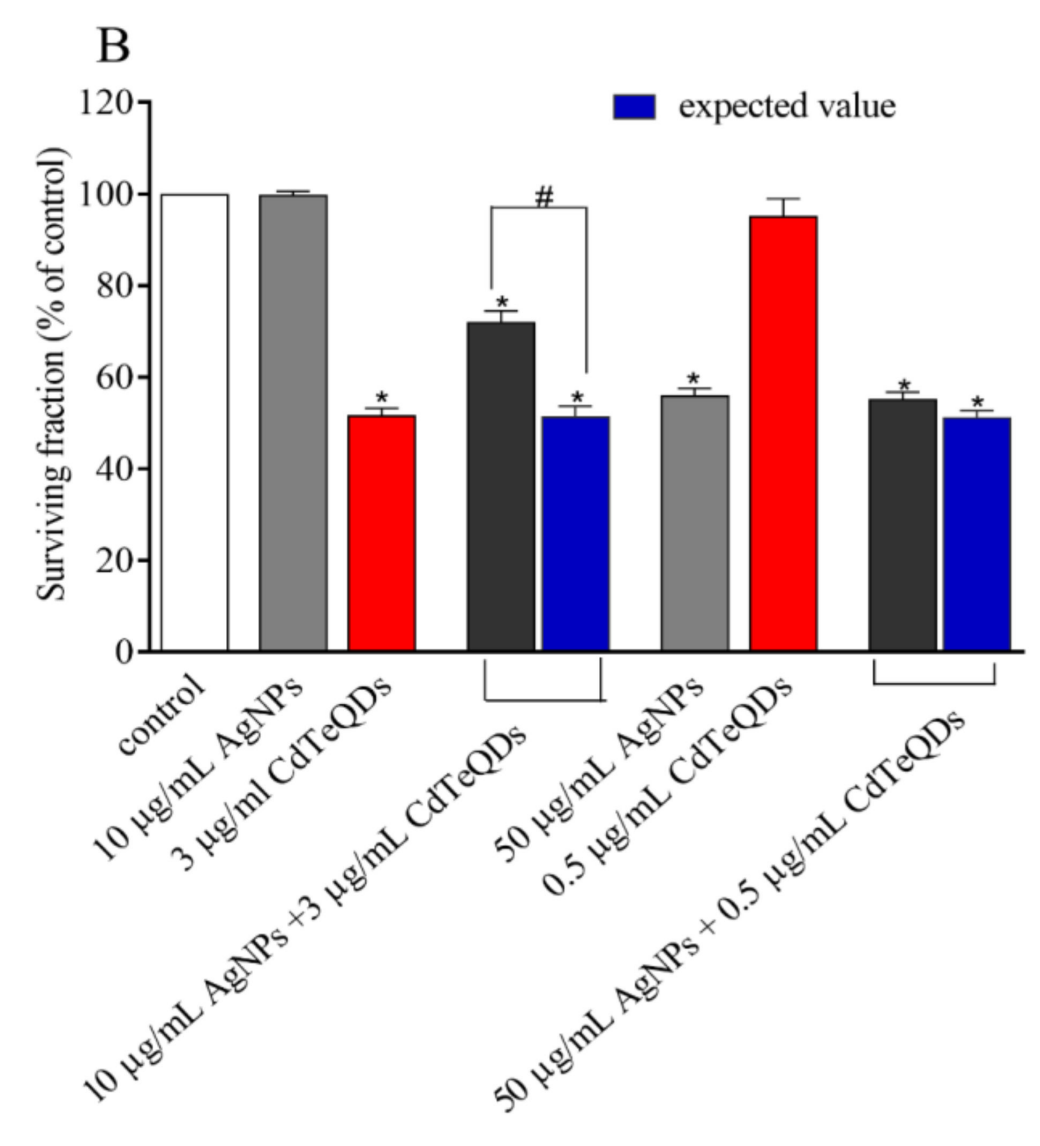

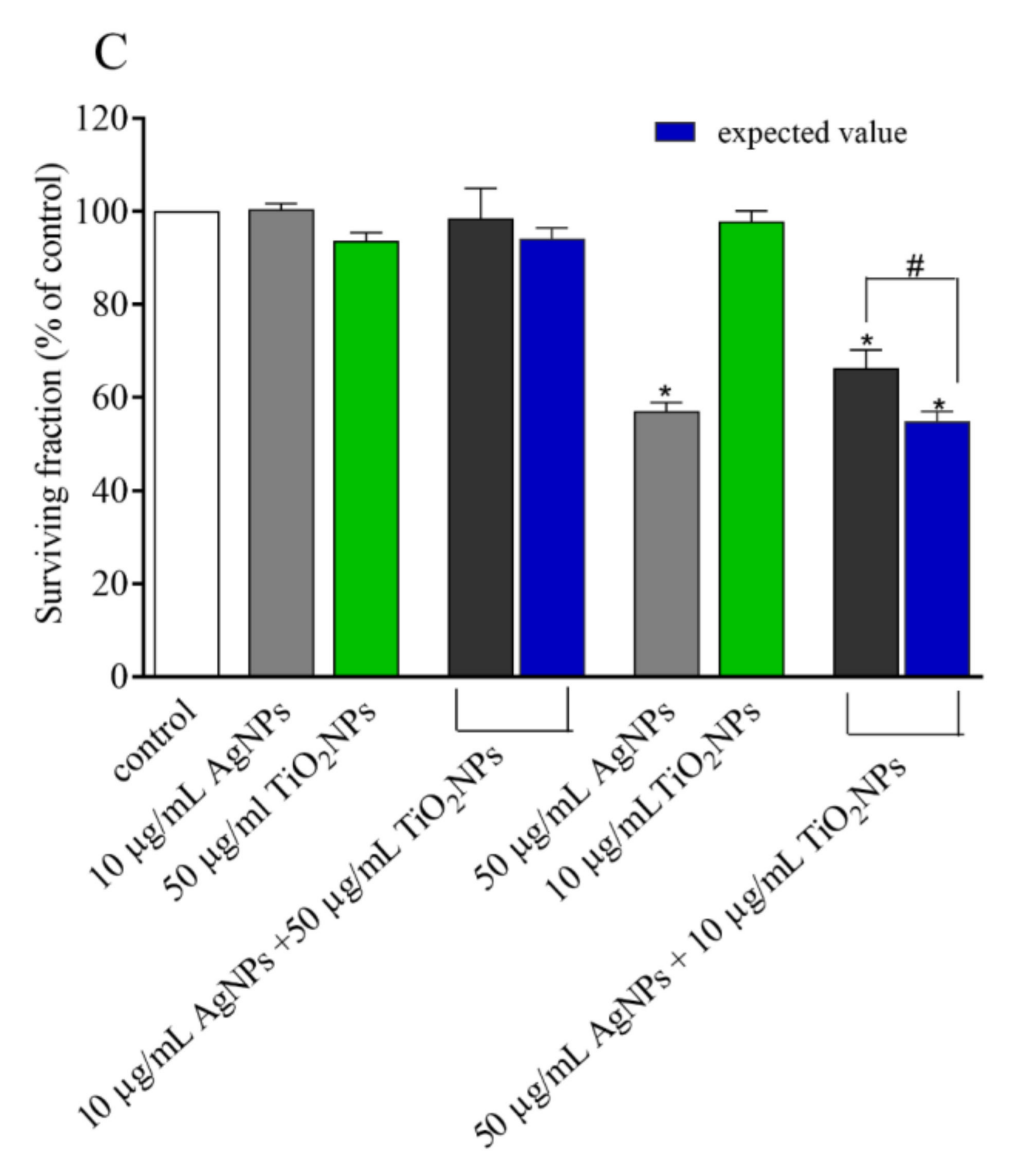

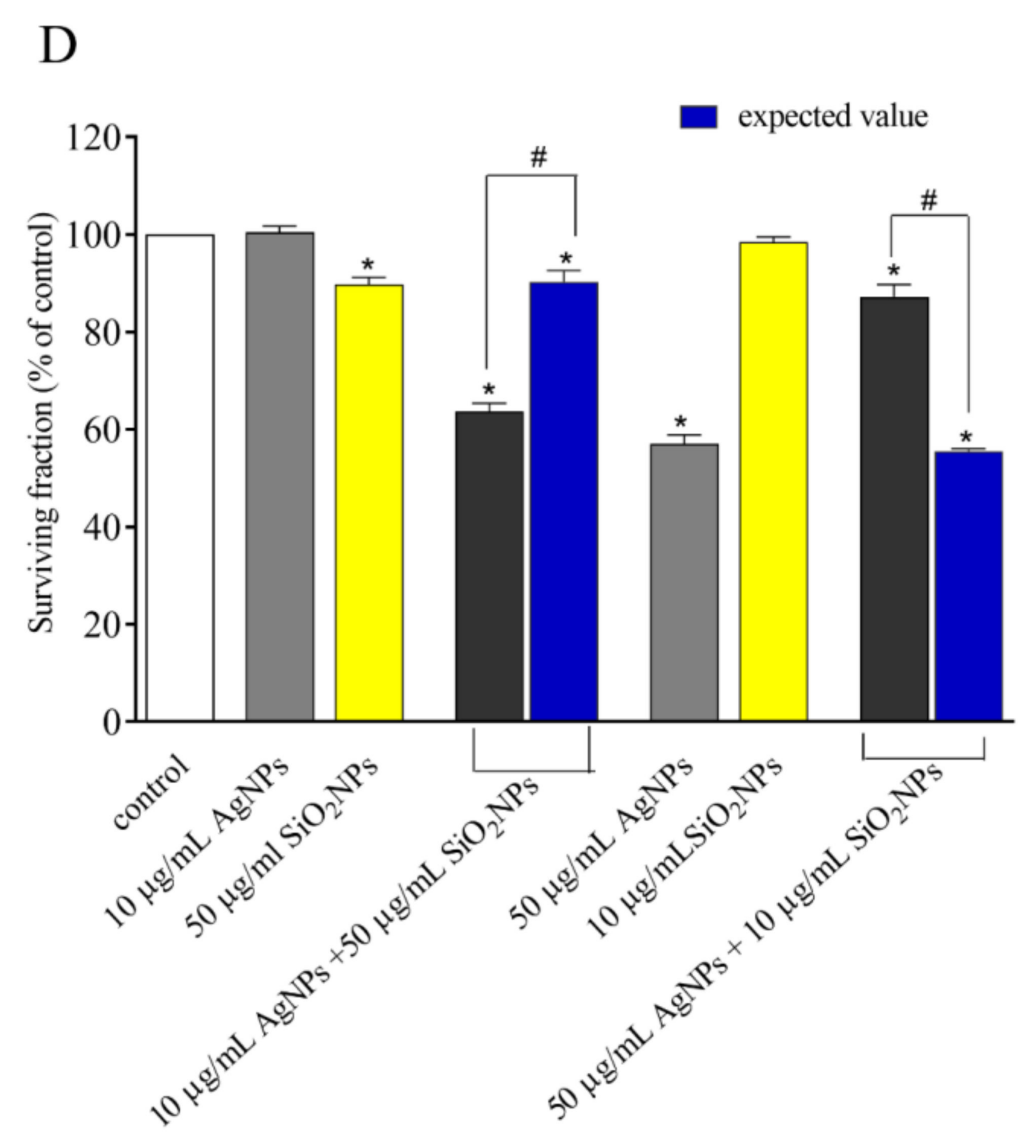
Viability (neutral red (NR) assay) of HepG2 cells treated with the combination of AgNPs with AuNPs (**A**), CdTeQDs (**B**), TiO_2_NPs (**C**), and SiO_2_NPs (**D**). Expected values reflect a sum of toxicities of AgNPs and other NPs (additive effect). Data are expressed as a percent of control; mean ± SD, n = 3. * denotes a statistically significant difference from unexposed control, *P* < 0.05. # denotes a statistically significant difference between the observed and expected value, *P* < 0.05.

The toxicity of AgNPs was further investigated in combinations with other NPs. We decided to apply two different regimes of treatment: (1) Low-AgNP-toxicity regime: AgNP in concentration of 10 µg/mL and other NPs in concentration of 50 µg/mL; or (2) high-AgNP-toxicity regime: AgNPs in concentration of 50 µg/mL and other NPs in concentration of 10 µg/mL. The only exception was the mixture AgNPs:CdTeQDs. Due to the high toxicity of CdTeQDs, their concentration was reduced to 0.5 or 3 µg/mL, respectively. As expected, the most toxic mixture was AgNPs:CdTeQDs. However, when the expressed toxicity of AgNP binary mixtures was compared to expected values (the sum of toxicities of both NPs alone that reflects additive effect), a protective effect could be seen. The treatment of cells with mixtures of NPs resulted in a significant increase in viability of HepG2 cells after 48 h of incubation, as compared to the sum of toxicities of individual NPs. Although the sparring effect of co-treatment was observed in the majority of combinations, two exceptions could be noted, namely (1) the combination of 10 µg/mL AgNPs with 50 µg/mL SiO_2_NPs (low-AgNP-toxicity regime) resulted in a significant decrease in the survival of the cells, suggesting a synergistic mode of action; whereas (2) the combination of 50 µg/mL AgNPs with 0.5 µg/mL CdTeQDs (high-AgNP-toxicity regime) resulted in equal toxicity, suggesting a simple additive effect. However, alternate treatments, i.e., the AgNPs:SiO_2_NPs combination in the high-AgNP-toxicity regime or AgNPs:CdTeQDs combination in the low-AgNP-toxicity regime resulted in the sparring effect observed for the treatments with other combinations (Figure 2).

### 3.4. ROS Induction

**Figure 3:**
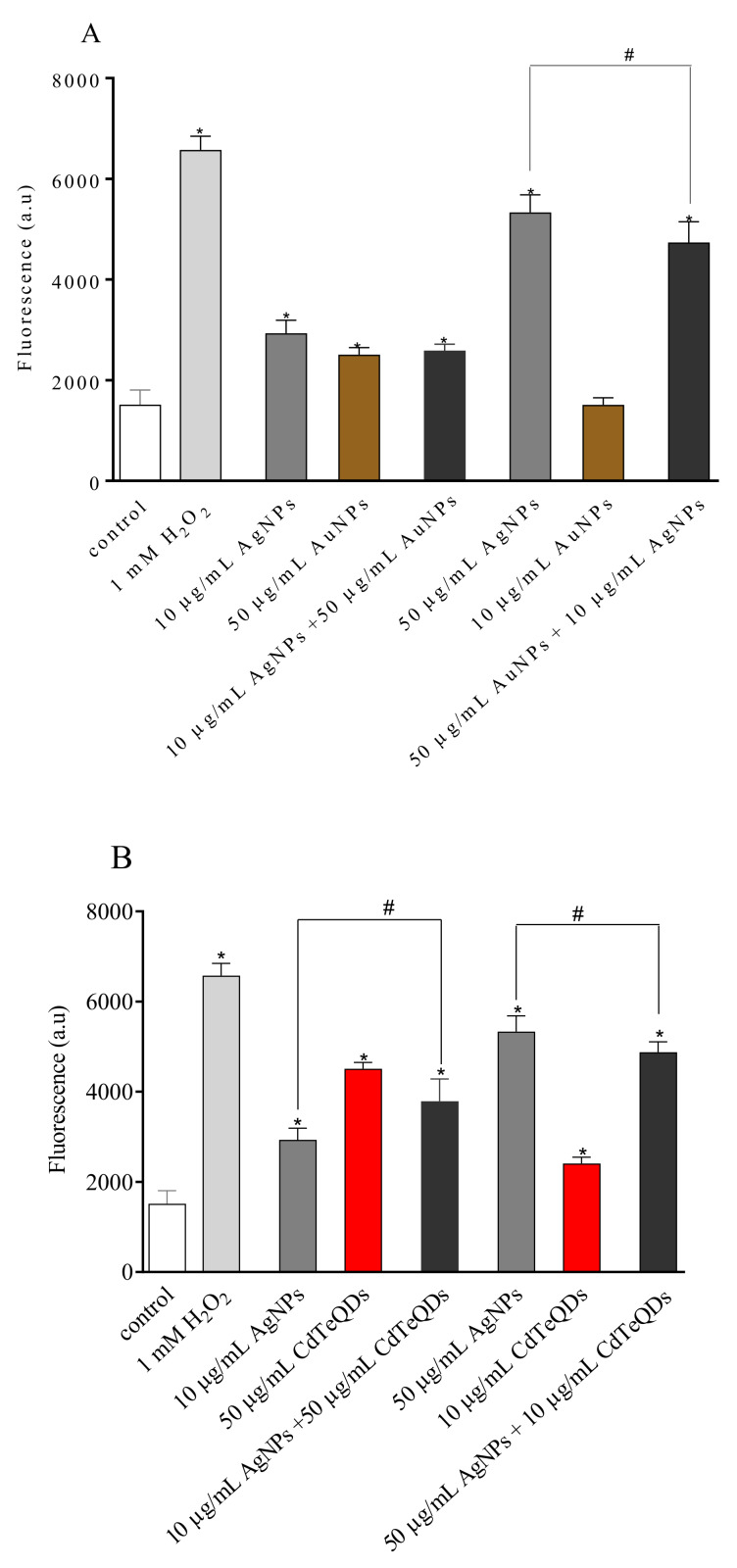

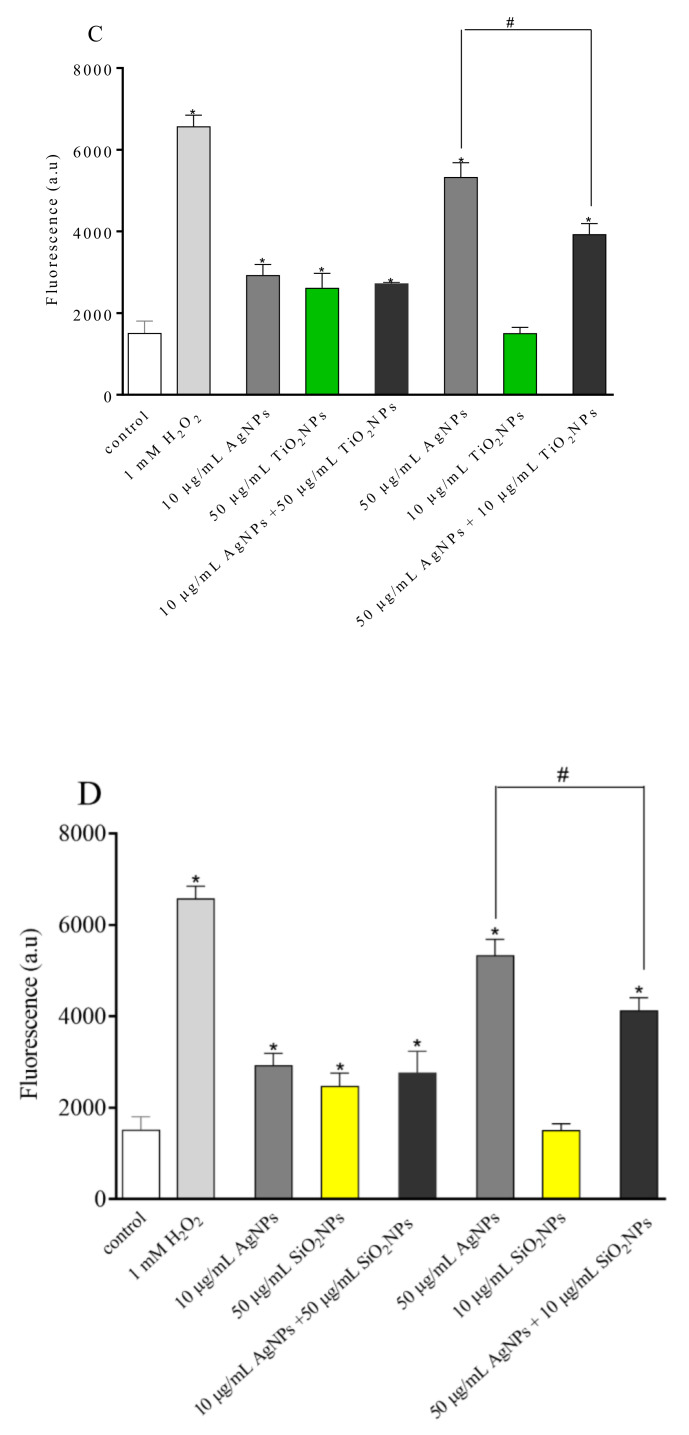
Generation of reactive oxygen species (ROS) in HepG2 cells treated with single NPs or the combination of AgNPs with AuNPs (**A**), CdTeQDs (**B**), TiO_2_NPs (**C**), and SiO_2_NPs (**D**). H_2_O_2_ in a concentration of 1 mM served as a positive control. Mean ± SD, n = 3. * denotes a statistically significant difference from the unexposed control, *P* < 0.05. # denotes a statistically significant difference between ROS generated by the combination of NPs and the highest value observed for the single NP in this combination, *P* < 0.05.

Several studies have provided strong evidence for a link between ROS production mediated by AgNPs and the subsequent generation of oxidative stress and cytotoxicity. Inline, our results except for AuNPs, TiO_2_NPs, and SiO_2_NPs in a concentration of 10 μg/mL indicate that all NPs induced a significant production of ROS (Figure 3). The highest production of ROS was observed for AgNPs and CdTeQDs. In addition, all tested NP binary mixtures induced a significant increase in ROS production in treated cells. However, except 50 μg/mL AgNPs + 10 μg/mL CdTeQDs treatment, all other treatments with the high-AgNP-toxicity regime resulted in a lower generation of ROS than the generation of ROS by 50 μg/mL AgNPs.

A significant difference in viability was observed between NP mixtures and the corresponding single exposure of NPs. The cell viability significantly increased for 50 µg/mL AgNPs co-exposed with 10 µg/mL AuNPs, as compared to a single treatment with 50 µg/mL AgNPs. The viability results of this mixture correlated well with the generation of ROS. Treatment of the cells with 50 µg/mL AgNPs and 10 µg/mL AuNPs resulted in a lower level of ROS. Similar results were observed for the 50 µg/mL AgNPs and 10 µg/mL TiO_2_ NPs mixture and for the 50 µg/mL AgNPs and 10 µg/mL SiO_2_ NPs mixture. A different situation was observed for the mixture of AgNPs and CdTeQDs. Despite the slight but significant decrease in ROS generation in the cells treated with the NPs mixture, the toxicity of the NPs mixture was as expected from the toxicities of single NPs.

### 3.5. Single-Cell Gel Electrophoresis

**Figure 4:**
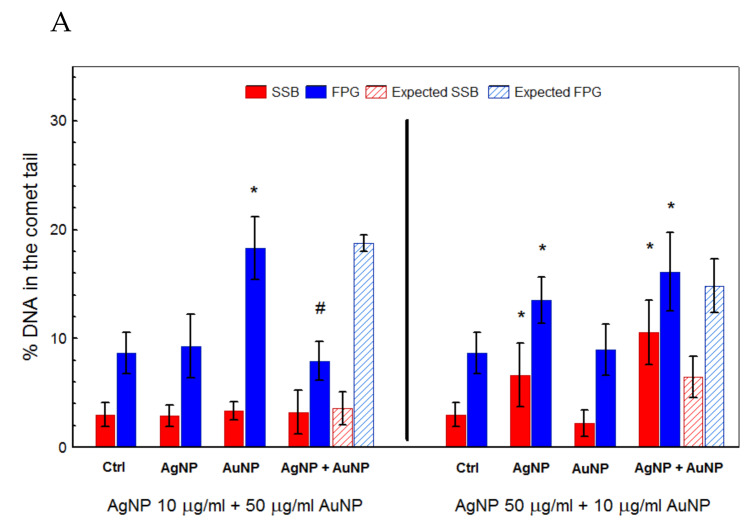

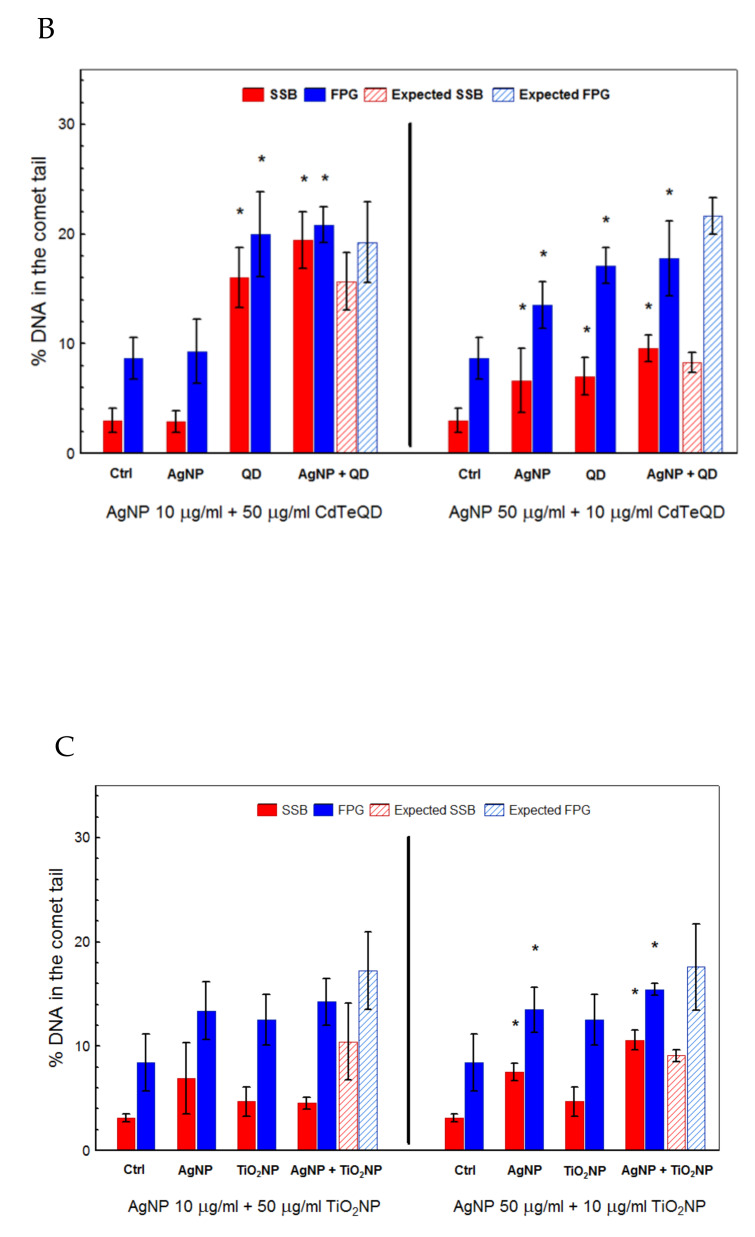

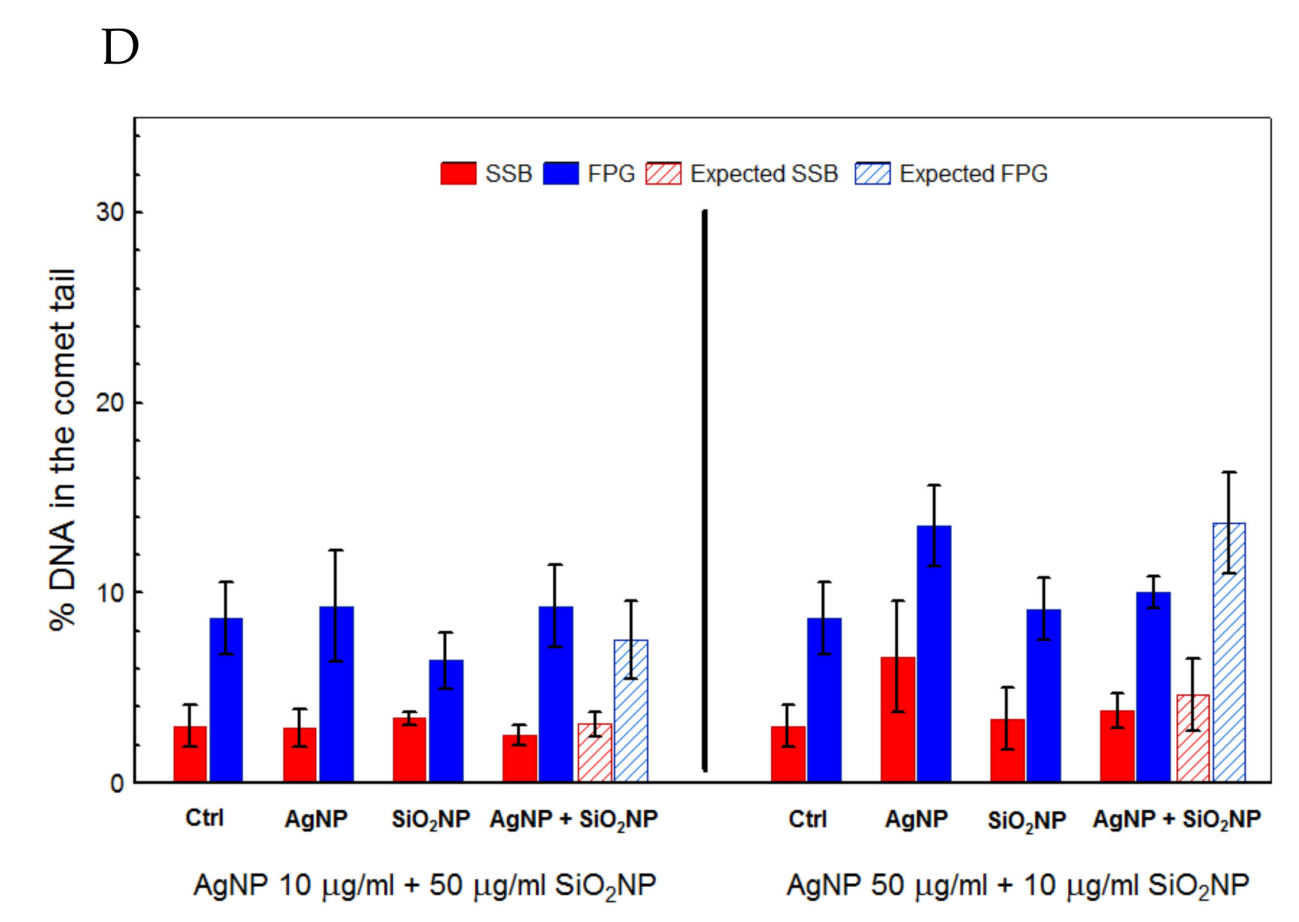
Induction of single-strand breaks (SSB) and base damage recognized by formamido-pyrimidine glycosylase (FPG) in HepG2 cells treated with the combination of AgNPs with AuNPs (**A**), CdTeQDs (**B**), TiO_2_NPs (**C**), and SiO_2_NPs **(D**) for 2 h. Mean ± SD, n = 3. * denotes a statistically significant difference from the unexposed control, *P* < 0.05. # denotes a statistically significant difference between observed and expected values, *P* < 0.05.

Studies using the comet assay showed that among the tested nanomaterials, CdTeQDs and AgNPs had the highest genotoxic potential. In the cells treated with AgNPs and CdTeQDs, a significant increase in the formation of DNA strand breaks was observed. At the same time, a significantly higher level of oxidative damage to DNA was also demonstrated for AuNPs. Analysis of the genotoxic potential of the NP mixtures showed simple additive effects, as DNA damage observed in cells treated with the NP mixture did not significantly differ from values expected for the sum of individual treatments. The exception was the treatment with the AgNPs + AuNPs mixture in the high-AgNP-toxicity regime, the high induction of oxidative damage to DNA observed for AuNPs alone was not confirmed for the AgNPs + AuNPs mixture, as oxidative damage to DNA found in the cells treated with this mixture did not differ from the control value (Figure 4).

## 4. Discussion

In a well-dispersed suspension, the average hydrodynamic diameter of a NP mixture should reflect the diameter of individual NPs, eventually shifted to the higher values due to the specificity of the DLS method [24]. However, the mixtures tested in this study had hydrodynamic values higher than those of individual NPs, reflecting the formation of NP agglomerates. Indeed, the formation of NP agglomerates in the mixture was confirmed by the increase in the polydispersity index (PDI), revealing the heterogeneity of the mixture (Table 1). Agglomeration of NPs usually corresponds to their zeta potential values, NPs with a zeta potential between +30 and –30 mV, showing a maximum [25]. Though this general rule also applies to our measurements, as the two most unstable NPs, namely AgNPs and CdTeQDs, had a zeta potential between –30 and 0 mV, TiO_2_NPs and SiO_2_NPs showed excellent stability despite the zeta potential in the same range (Table 2). This suggests additional factors that might affect the NP suspension stability. One of these factors could be interactions between protein corona on the NPs’ surface, as it was shown that NPs with different zeta potentials attract differently [26]. 

The zeta potential rule was, however, supported by the measurement of the zeta potential of NP mixtures. All studied binary mixtures except for the AgNPs:SiO_2_NPs mixture showed a zeta potential less than –30 mV, characterized with moderate stability. For the majority of mixtures, it was more or less an average of potentials of the individual NPs; however, the zeta potential of the mixture of AgNPs:TiO_2_NPs was higher than the zeta potential of the individual NPs. As the zeta potential roughly reflects a charge on the NP surface that depends on the composition of the protein corona, our results may indicate a different affinity to the cell culture medium proteins of the AgNPs:TiO_2_NPs mixture and AgNPs or TiO_2_NPs alone (Table 3).

The NP size, zeta potential, and composition of protein corona directly affect their uptake by cells [27]. Indeed, the cells took larger aggregates present in NP binary mixtures to a much lesser extent than the smaller aggregates of single NPs (Table 1). This was easily observed in the case of the quickly aggregating AgNPs+TiO_2_NPs mixture; however, even a small change in the size of aggregates observed for the AgNPs+SiO_2_NPs mixture resulted in their smaller uptake (Table 1, Figure 1). Moreover, as NPs share similar mechanisms for entry into the cell, the NPs present in the mixtures might compete for available entry routes. In all tested cases, uptake of NPs in the mixture was significantly lower than that expected for the sum of NPs alone (Figure 1).

In recent years, it has become clear that NPs uptake and ROS production determine their cyto- and genotoxicity [28]. Our results seem to support this assumption. Except for the mixture, 10 µg/mL AgNPs with 50 µg/mL SiO_2_NPs (synergistic effect) and 50 µg/mL AgNPs with 0.5 µg/mL CdTeQDs (additive effect), the toxicity of other NP binary mixtures was lower than that expected from the sum of toxicities of NPs alone (sparring effect). This suggests that NPs also share mechanisms of cell killing. Indeed, ROS production was pointed out as the primary mechanism of NPs [21,28]. In line with the toxicity pattern, production of ROS by cells treated with NP mixtures was lower than that of the highest value observed for the single NP in this combination (Figure 3).

Interestingly, decreased uptake and ROS production did not affect the ability of NP mixtures to induce damage to DNA. Except for 10 µg/mL AgNPs with 50 µg/mL AuNPs, all treatments with NP mixtures resulted in an induction of DNA single-strand breaks and Fpg-sensitive sites at the level expected for the sum of damage induced by corresponding NPs alone (Figure 4), suggesting a simple additive effect. It seems that it must be another intracellular factor that limits NP-dependent induction of damage to DNA. It has been proposed recently that Fenton reaction-dependent ROS production is a crucial mechanism of AgNPs [29]. If this is so, although decreased uptake of NPs in the mixture resulted in decreased ROS production, the production of harmful OH radicals is limited by the availability of iron ions, not by H_2_O_2_ production by NP-induced damage to mitochondria. Thus, if OH radical production by the decreased amount of NPs saturated the Fenton reaction capacity, a further increase in ROS production (H_2_O_2_) does not elevate the production of OH radicals.

Despite an increasing number of studies demonstrating the benefits of nanomaterials in everyday life, little is known about the risks they pose to human health and the environment. The results obtained herein are of great importance from the point of view of general toxicology and public health, improving our understanding of the impact of NPs on biological systems and helping in better estimating the risk associated with their use. On the other hand, our results might also be interesting from the medico-pharmaceutical point of view. Our results clearly showed that the biological effects of a particular type of NPs could be changed by the addition of different types of NPs; thus, undesired side-effects of NP applications could be diminished by appropriate co-treatment with other NPs.

## 5. Conclusions

Despite many studies on the toxicity of nanoparticles, there is little information on the effects of nanoparticle mixtures on the environment and humans. Our results show that the toxicity of nanoparticles in mixtures may differ from that observed for single nanoparticles. Furthermore, the toxicity induced by a single nanoparticle may be modified (increased or decreased) by co-exposure to nanoparticles with different compositions. Our results reflect the complexity of the response to the co-exposure of different NPs. Different types of NPs can interact in different ways; thus, it is difficult to predict the cellular response just based on the biological effects of single particles.

These results are relevant for the risk assessment of human exposure to nanoparticles. Potential co-exposure with other nanomaterials could be useful to diminish possible toxic effects or even protect against inflammation.

## Figures and Tables

**Table 1:** Hydrodynamic diameter and zeta potential of nanoparticles (NPs) alone or their mixtures.

Nanoparticle/ Nanoparticle Mixture	Hydrodynamic Diameter	PDI	Zeta Potential
**AgNPs**	86 ± 2	0.317 ± 0.04	–28.3 ± 0.5
**AuNPs**	25 ± 1	0.135 ± 0.02	–45.6 ± 0.5
**AgNPs:AuNPs**	95 ± 3	0.420 ± 0.05	–35.7 ± 2.5
**CdTeQDs**	96 ± 2	0.480 ± 0.07	–10.5 ± 0.2
**AgNPs: CdTeQDs**	158 ± 2	0.535 ± 0.05	–45.6 ± 1.1
**TiO_2_NPs**	246 ± 1	0.193 ± 0.07	–25.5 ± 0.6
**AgNPs: TiO_2_NPs**	446 ± 6	0.598 ± 0.10	–37.2 ± 2.6
**SiO_2_NPs**	124 ± 6	0.380 ± 0.02	–20.2 ± 2.0
**AgNPs: SiO_2_NPs**	187 ± 33	0.452 ± 0.02	–25.8 ± 4.8

Data are expressed as the mean ± SD, n = 3.

**Table 2:** Agglomeration of NPs alone.

NPs	Time	Hydrodynamic Diameter (nm)	PDI
**Ag**	*0 min*	86 ± 2	0.317 ± 0.04
	*15 min*	98 ± 5	0.288 ± 0.07
	*30 min*	153 ± 4	0.262 ± 0.10
	*60 min*	158 ± 2	0.276 ± 0.02
	*120 min*	151 ± 5	0.279 ± 0.08
**Au**	*0 min*	25 ± 1	0.135 ± 0.02
	*15 min*	28 ± 2	0.145 ± 0.07
	*30 min*	29 ± 2	0.137 ± 0.05
	*60 min*	26 ± 2	0.130 ± 0.01
	*120 min*	28 ± 3	0.141 ± 0.05
**CdTeQDs**	*0 min*	96 ± 2	0.480 ± 0.07
	*15 min*	149 ± 8	0.450 ± 0.05
	*30 min*	148 ± 10	0.505 ± 0.07
	*60 min*	150 ± 6	0.489 ± 0.12
	*120 min*	159 ± 3	0.493 ± 0.07
**TiO_2_**	*0 min*	246 ± 1	0.193 ± 0.07
	*15 min*	242 ± 5	0.203 ± 0.04
	*30 min*	250 ± 3	0.196 ± 0.01
	*60 min*	244 ± 3	0.194 ± 0.23
	*120 min*	241 ± 1	0.173 ± 0.08
**SiO_2_**	*0 min*	124 ± 6	0.380 ± 0.02
	*15 min*	133 ± 7	0.340 ± 0.06
	*30 min*	127 ± 4	0.300 ± 0.12
	*60 min*	140 ± 4	0.360 ± 0.09
	*120 min*	139 ± 1	0.290 ± 0.04

**Table 3:** Agglomeration of NP mixtures.

NPs Mixture*	Time	Hydrodynamic Diameter (nm)	PDI
**Ag:Au**	*0 min*	95 ± 3	0.420 ± 0.05
	*5 min*	106 ± 5	0.320 ± 0.03
	*30 min*	129 ± 8	0.410 ± 0.05
	*60 min*	126 ± 8	0.420 ± 0.01
	*120 min*	117 ± 8	0.410 ± 0.5
**Ag:CdTeQDs**	*0 min*	158 ± 6	0.535 ± 0.05
	*15 min*	167 ± 8	0.550 ± 0.05
	*30 min*	189 ± 6	0.535 ± 0.05
	*60 min*	158 ± 8	0.589 ± 0.02
	*120 min*	172 ± 6	0.593 ± 0.07
**Ag:TiO_2_**	*0 min*	446 ± 6	0.598 ± 0.10
	*15 min*	450 ± 9	0.670 ± 0.05
	*30 min*	636 ± 10	0.660 ± 0.02
	*60 min*	685 ± 6	0.610 ± 0.06
	*120 min*	625 ± 7	0.730 ± 0.01
**Ag:SiO_2_**	*0 min*	187 ± 3	0.452 ± 0.02
	*15 min*	191 ± 2	0.430 ± 0.01
	*30 min*	211 ± 9	0.470 ± 0.01
	*60 min*	246 ± 3	0.465 ± 0.01
	*120 min*	253 ± 5	0.468 ± 0.03

* - Agglomeration of NP mixtures was measured using a 1:1 ratio (10 µg/ml AgNPs:10 µg/ml another NPs).

## Supplementary Materials

The following are available online at https://www.mdpi.com/1996-1944/13/10/2221/s1, Figure S1: Toxicity of Au, Ag, TiO_2_, and SiO_2_ nanoparticles and CdTe quantum dots (reprinted from doi: 10.26444/aaem/120664 with permission). Figure S2: Uptake/binding of CdTe quantum dots single or combination with AgNPs (histograms).
