# Supplementary figures and images for: Susceptibility of HepG2 Cells to Silver Nanoparticles in Combination with other Metal/Metal Oxide Nanoparticles
Source: Materials (Basel). 2020 May 12;13(10):2221. doi: 10.3390/ma13102221 (PMC7287770; doi:10.3390/ma13102221)

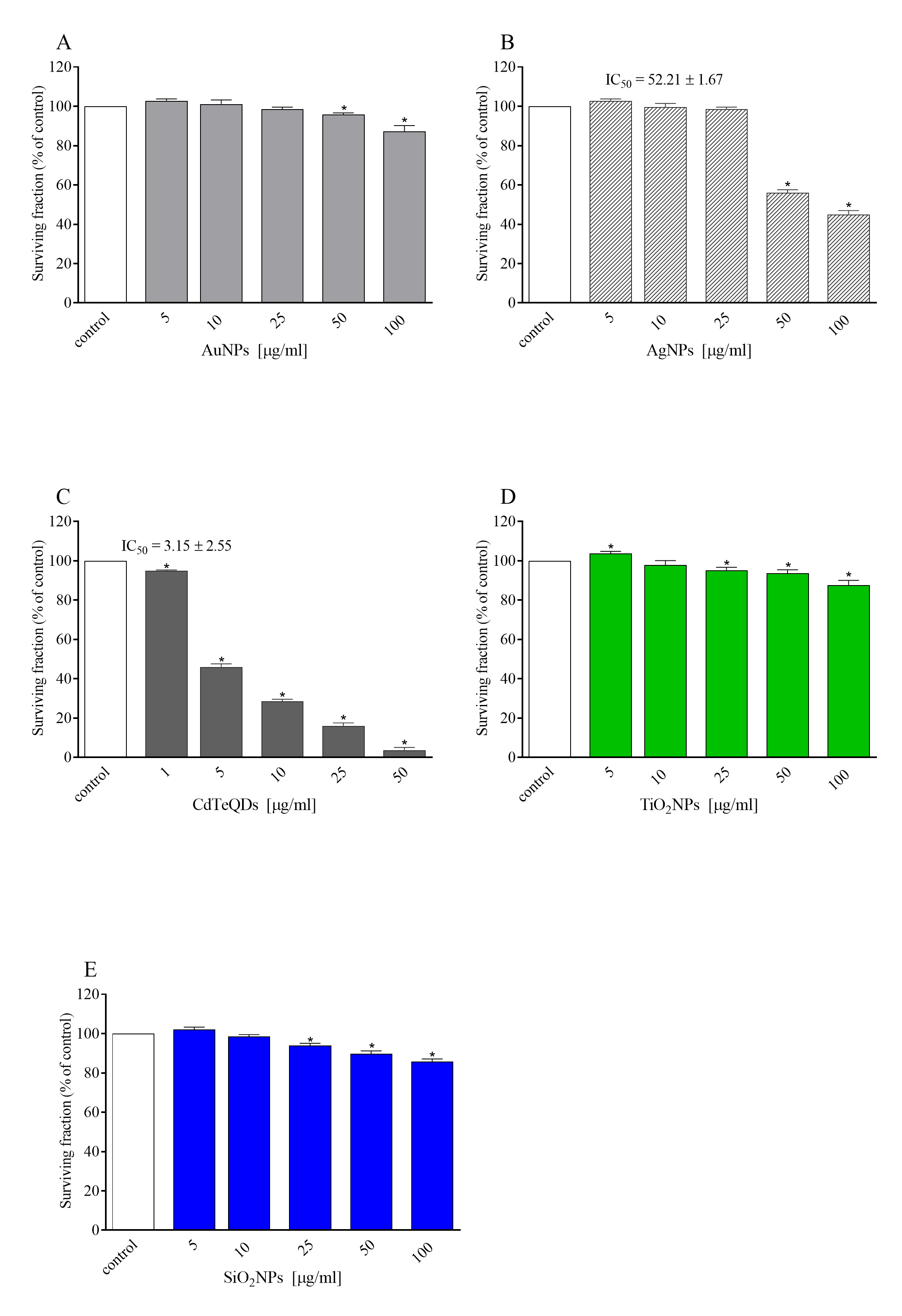

Supplement: Supplementary file 1 [file materials-13-02221-s001.zip › Supplementary Figure 1.tif]

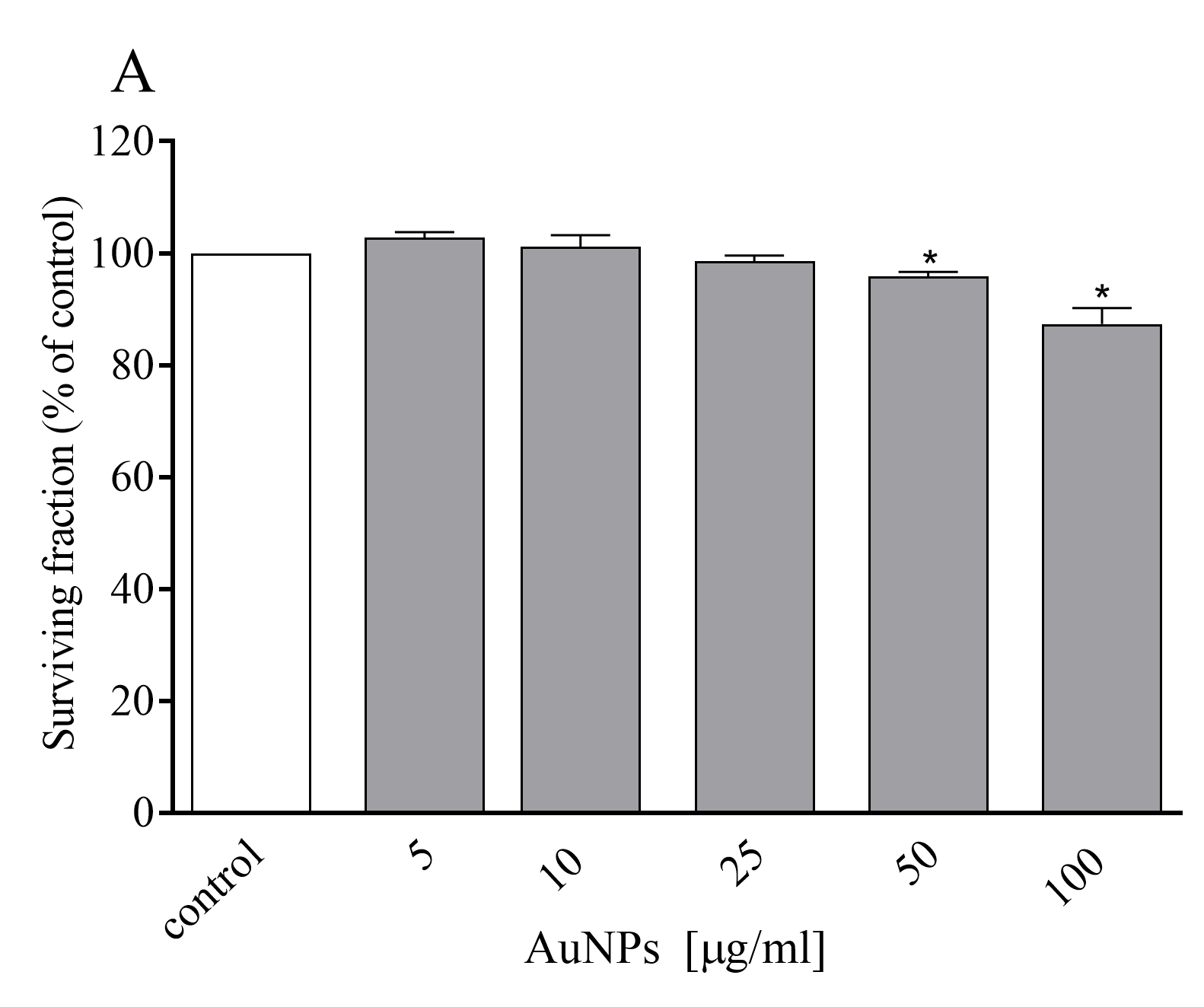

Supplement: Supplementary file 1 [file materials-13-02221-s001.zip › Supplementary Figure 1A.tif]

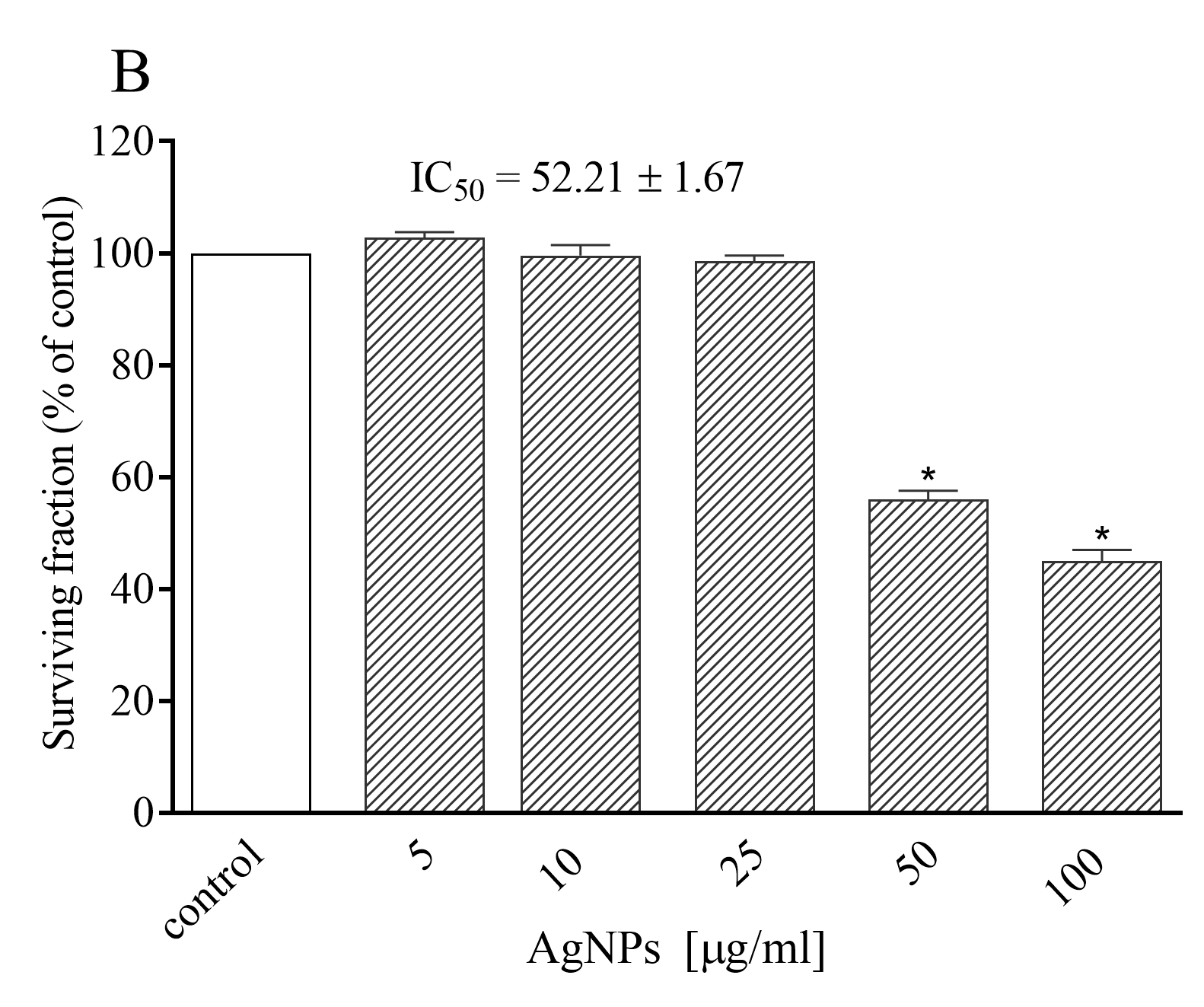

Supplement: Supplementary file 1 [file materials-13-02221-s001.zip › Supplementary Figure 1B.tif]

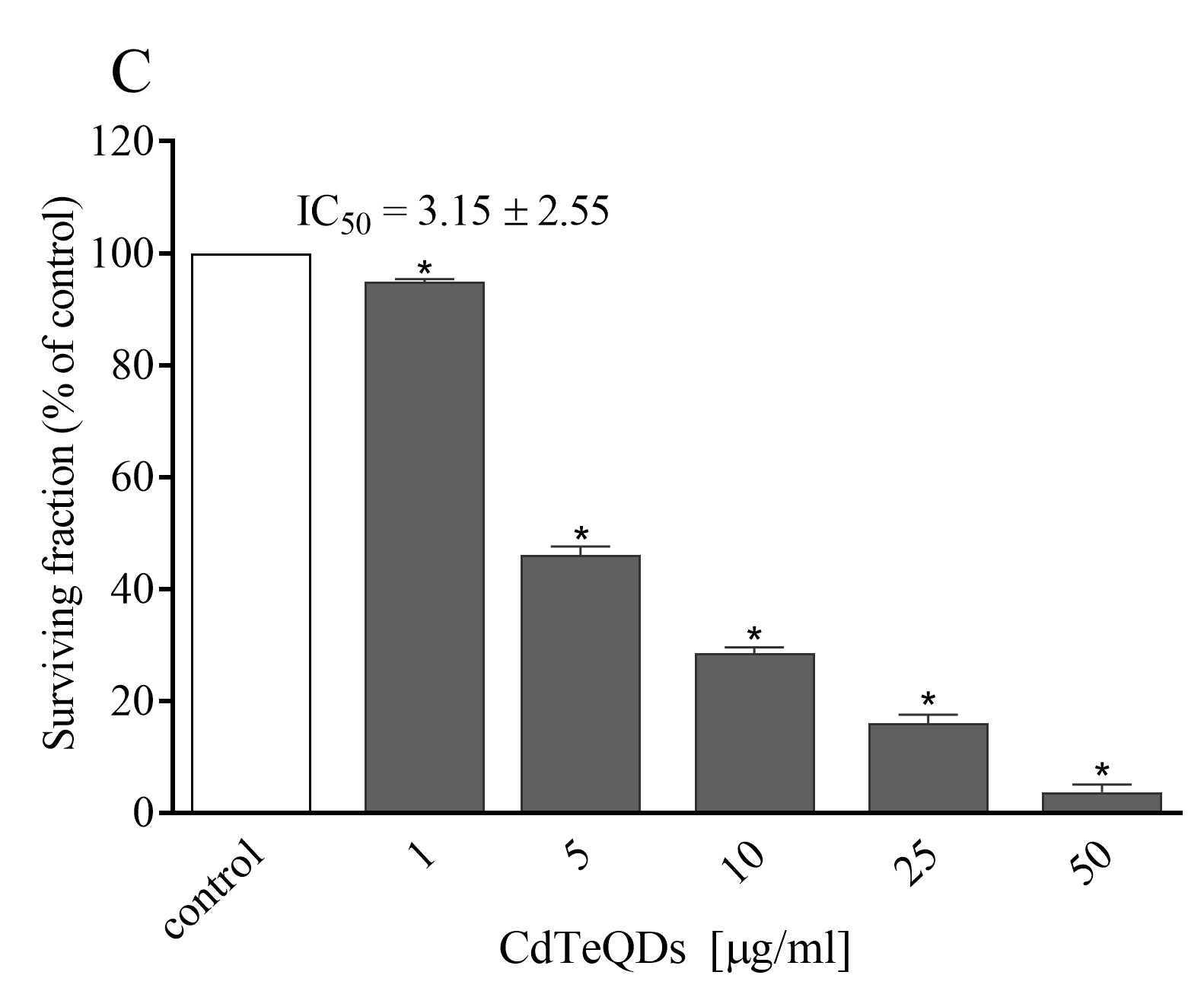

Supplement: Supplementary file 1 [file materials-13-02221-s001.zip › Supplementary Figure 1C.tif]

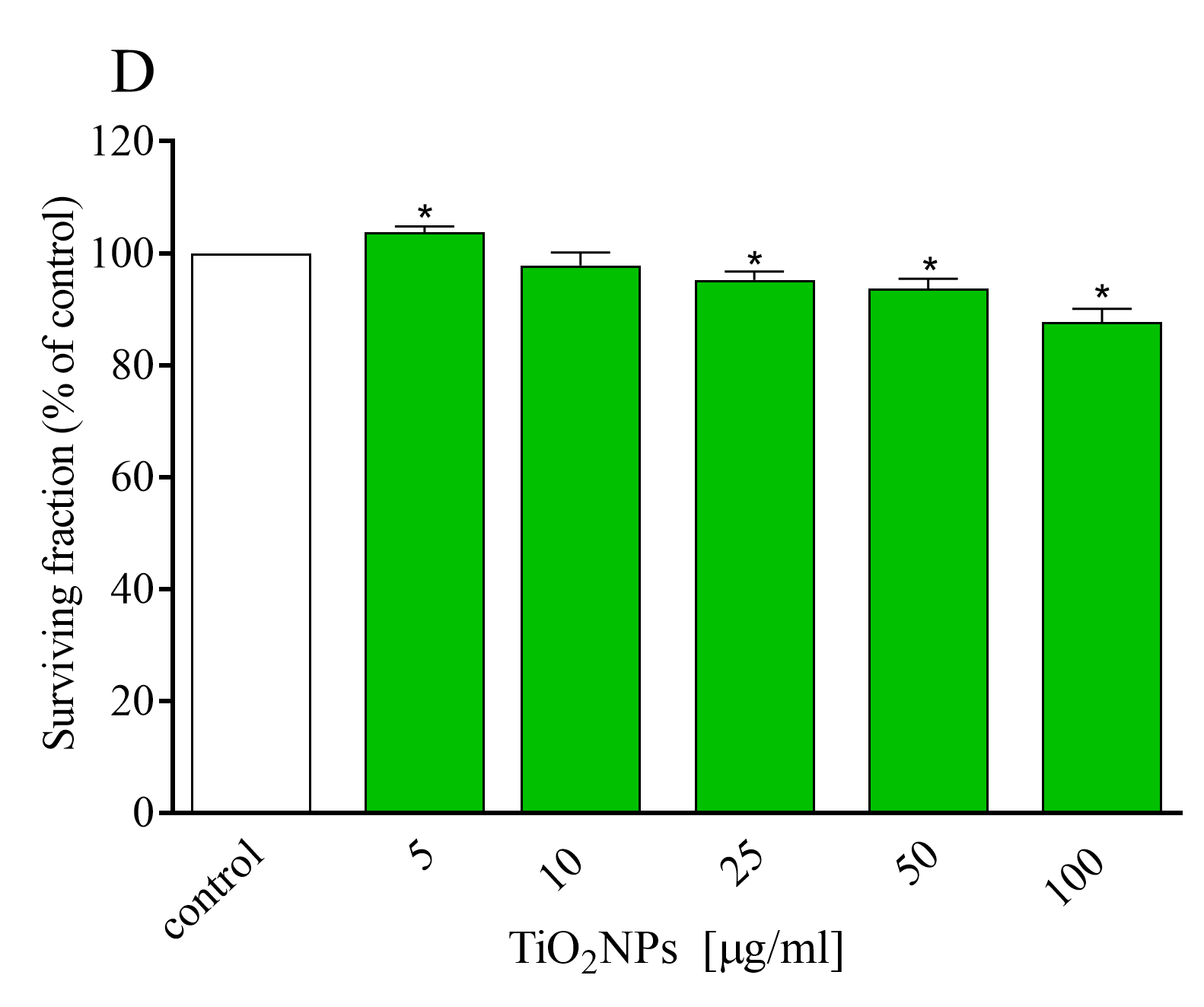

Supplement: Supplementary file 1 [file materials-13-02221-s001.zip › Supplementary Figure 1D.tif]

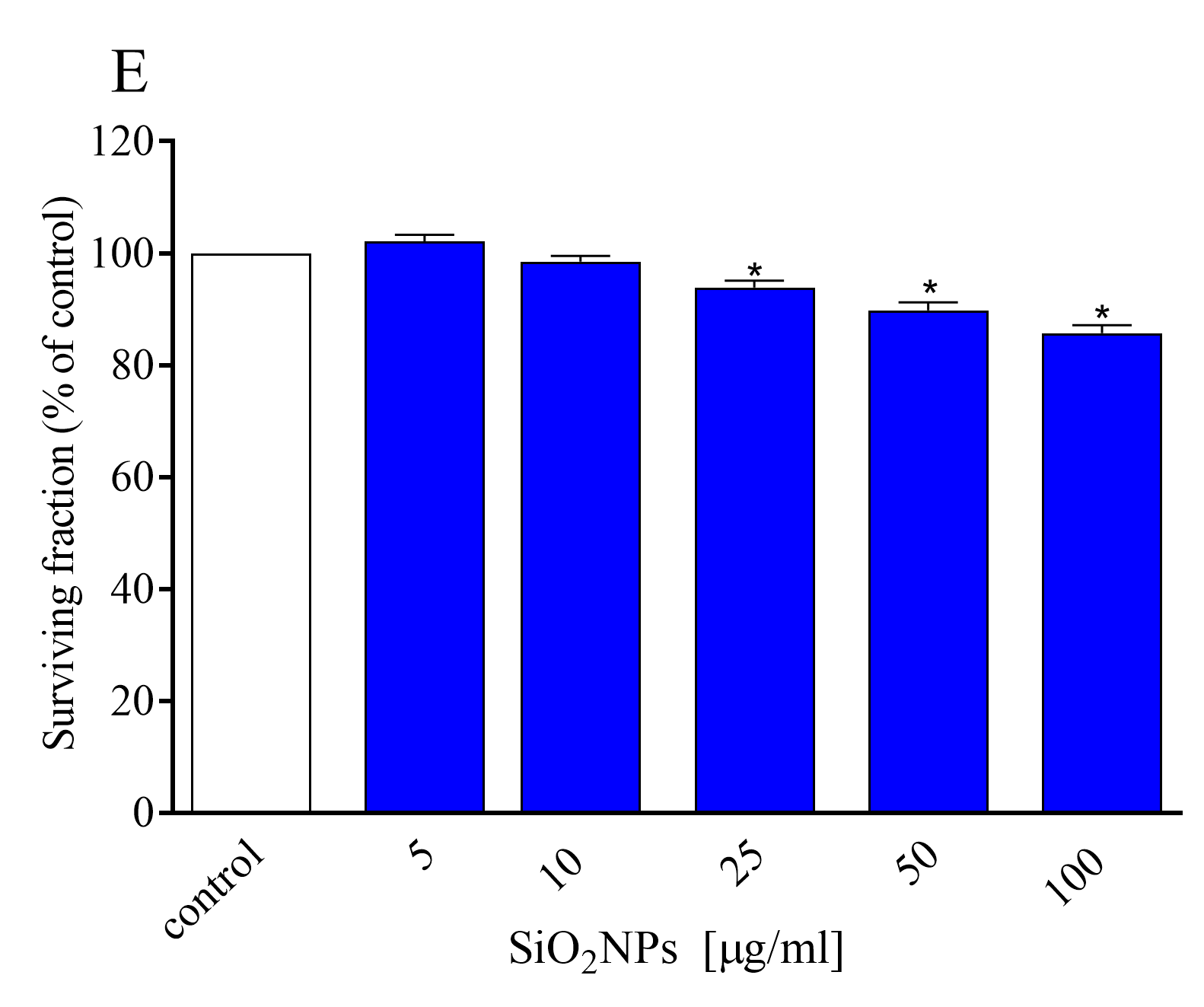

Supplement: Supplementary file 1 [file materials-13-02221-s001.zip › Supplementary Figure 1E.tif]

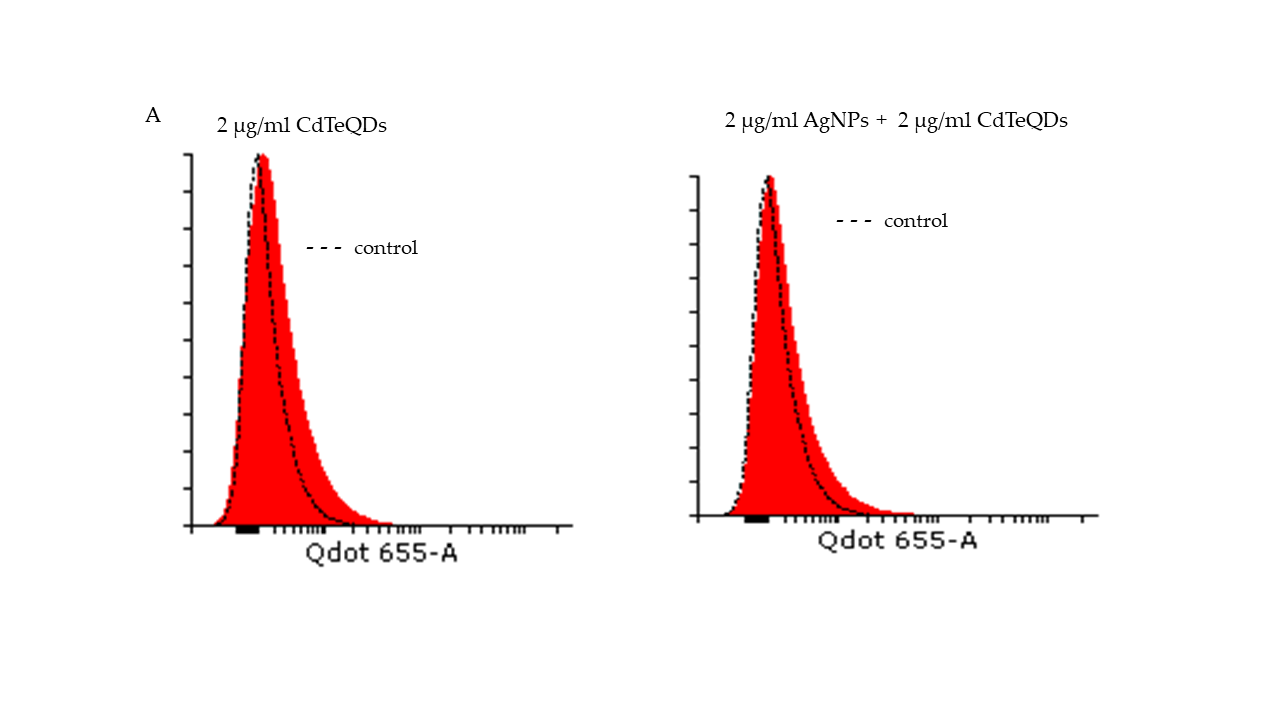

Supplement: Supplementary file 1 [file materials-13-02221-s001.zip › Supplementary Figure 2A.TIF]

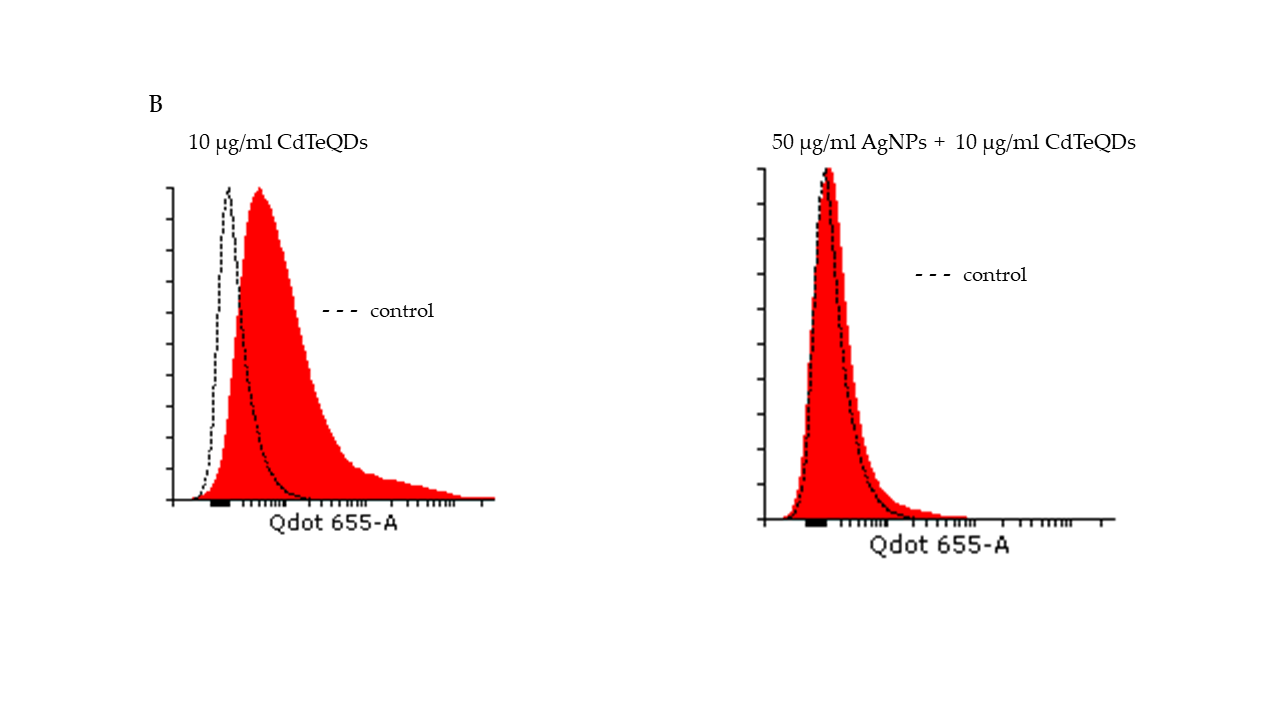

Supplement: Supplementary file 1 [file materials-13-02221-s001.zip › Supplementary Figure 2B.TIF]

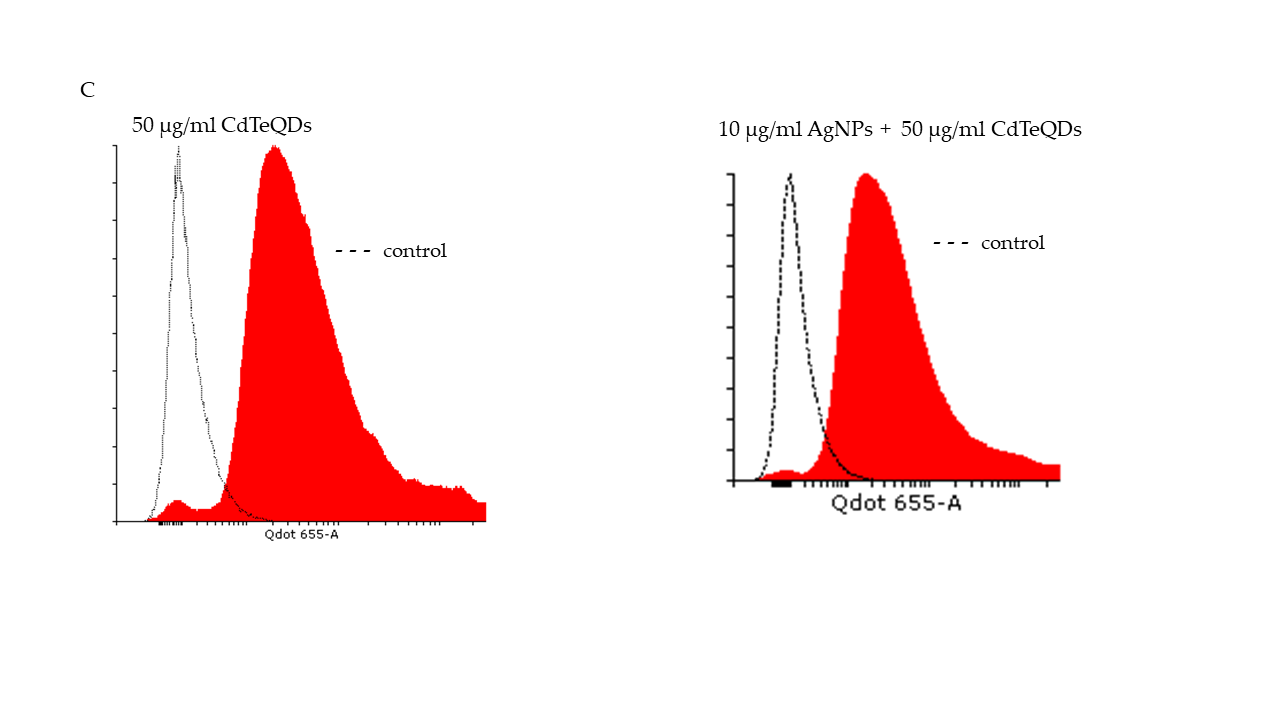

Supplement: Supplementary file 1 [file materials-13-02221-s001.zip › Supplementary Figure 2C.TIF]
